# Protein interactors of Spindle Pole Body (SPB) components and septal proteins in fungus *Neurospora crassa*: A mass spectrometry-based dataset

**DOI:** 10.1016/j.dib.2023.109980

**Published:** 2024-01-01

**Authors:** Astrid N. Espino-Vázquez, Rosa Ramírez Cota, Olga Alicia Callejas-Negrete, Reinhard Fischer, Rosa R. Mouriño-Pérez

**Affiliations:** a Departamento de Microbiología. Centro de Investigación Científica y de Educación Superior de Ensenada (CICESE). Ensenada, B.C., Mexico; b Department of Microbiology, Institute for Applied Biosciences, Karlsruhe Institute of Technology (KIT)- South Campus, Fritz-Haber-Weg 4, Karlsruhe D-76131, Germany

**Keywords:** Co-immunoprecipitation, Protein-protein interactions, LC-MS/MS, Neurospora crassa, MTOC, γ-Tubulin, Spindle Pole Body

## Abstract

Microtubule Organizing Centers (MTOC) are subcellular structures in eukaryotic cells where nucleation of microtubules (MTs) takes place and represents the filament's minus end. Their localization depends on the species, cell type, and cell cycle stage. Along the fungal kingdom, the Spindle Pole Body (SPB) in the nucleus (an equivalent to Centrosomes in animal cells) is the principal MTOC. Other MTOCs have been identified in filamentous fungi, such as the Spitzenkörper in the hyphal tips of *Schizosaccharomyces pombe* or the septal pore of *Aspergillus nidulans*. However, in the fungal-model organism *Neurospora crassa*, these alternative MTOCs have not been recognized. Here, we present a Mass spectrometry-based dataset of proteins interacting with four MTOC components of *N. crassa* tagged with fluorescent proteins: γ-Tubulin-sGFP (main nucleator at the SPB), MZT-1-sGFP (structural SPB microprotein), APS-2-dRFP (septal protein and recognized SPB component), and SPA-10-sGFP (septal MTOC protein). A WT and a cytosolic GFP expressing strain were included as controls. The protein interactors were pulled down by Co-IP^1^, using GFP-Magnetic agarose that captures recombinant GFP proteins (including GFP-derivatives) in their native state. Bounded proteins were separated by SDS-PAGE and identified by nano LC-MS/MS^2^. The protein annotation was done using the *N. crassa* protein database.

Specifications Table

**Table:** 

Subject	*Biological sciences*
Specific subject area	*Fungal Cell Biology*
Data format	RAW files of Mass spectrometry (.raw) Analyzed and filtered in Excel files (.xlsx)
Type of data	Raw data, Table, Figure
Data collection	Instruments: Ultimate 3000 nano UHPLC system with a trapping column (PepMap C18, 100 Å, 100 µm × 2 cm, 5 µm) and an analytical column (PepMap C18, 100 Å, 75 µm × 50 cm, 2 µm). Q Exactive HF mass spectrometer (Thermo Fisher Scientific, USA) with an ESI nanospray source. Protocol: Total native proteins from six strains of *N. crassa* were extracted and incubated with GFP-Magnetic agarose pearls to trap fluorescent-labeled proteins and their interactors. The beads were separated in a magnetic rack, and, after five washes, bound proteins were suspended in a 2x SDS-Sample buffer. Eluted proteins were electrophoresed in SDS-PAGE and stained with Coomassie blue. Selected gel lanes were cut and sent for protein identification service, where the in-gel digestion with trypsin, peptide extraction, desalting, and LC-MS/MS analysis were performed. The *N. crassa* database was used for protein annotation.
Data source location	Centro de Investigación Científica y de Educación Superior de Ensenada (CICESE). Ensenada, B.C., México. Coordinates 31.8675° N, 116.6652° W
Data accessibility	Espino-Vazquez, Astrid N; Ramírez Cota, Rosa; Callejas-Negrete, Olga Alicia; Mouriño-Pérez, Rosa (2022), “Identified protein interactors of four predicted Microtubule Organizing Center (MTOC) components in *N. crassa* by LC-MS/MS ”, Mendeley Data, V2, DOI: 10.17632/wyhbwxmykv.2.
Related research article	R. Ramírez-Cota, A.N. Espino-Vázquez, T.C Rodríguez-Vega, R.E. Macias-Díaz, O.A. Callejas-Negrete, M. Freitag, R. Fischer, R.W. Roberson, and R.R. Mouriño-Pérez, *N. crassa* has two kinds of MTOCs, Fungal Genetics and Biology, In Press

## 1 Value of the Data

• The raw data can be re-analyzed with a different method and search parameters to output new peptide search, protein inference, or protein concentration comparison. For example, the relative concentration of fusion proteins can be determined based on GFP quantification.
• Results files contain the mass spectrometry report of identified peptides and proteins with their relative protein abundance in the sample (intensity) and a score (confidence of identified results). According to the protein identification service provider, *a* > 50 score denotes high-confident results.
• Filtered lists display an extensive list of putative interactors (> 1000 per experiment) for γ-tubulin, MZT-1, APS-2, and SPA-10 proteins. Exploring these results opens the opportunity to explore novel physical interactions of those targets *in vitro* and *in vivo*.
• The whole dataset can be further exploited for researchers interested in studying MT assembly, SPB composition, or septal dynamics in *N. crassa* and other filamentous fungi.
• The control experiments (WT and cytosolic GFP) can also detect spurious data in equivalent experimental conditions.

## 2 Data Description

Here, we present a data set of proteins and peptides identified by Mass Spectrometry after Co-IP experiments. The Co-IP was carried out using native protein extracts of six recombinant *N. crassa* strains incubated with GFP-Trap Magnetic agarose. The files with the raw extension are MS data obtained by Nano LC-MS/MS Analysis. The Excel files contain the report of the characterized peptides using the *N. crassa* protein database.

• **1_WT.raw** file provides data from a negative control consisting of native proteins of a WT strain (FGSC 4200) bound to GFP-Trap magnetic beads under native conditions.
• **2_GFP.raw** file provides MS data from a positive control consisting of sGFP bound to GFP-Trap magnetic beads under native conditions. The crude extracts of the *N. crassa* strain expressing the sGFP in the cytosol were used for this control.
• **3_TUB-GFP.raw, 4_MZT-GFP.raw, 5_RFP-APS2.raw**, and **6_SPA10-GFP.raw** files are output MS files from experiments where the native extracts of *N. crassa* strains expressing fusion proteins were incubated with GFP-Trap magnetic beads under native conditions.
• **Results_1_WT.xlsx** is the MS report with annotated *N. crassa* proteins detected in the negative control (crude extracts of FGSC 4200 strain). These proteins are managed as unspecific physical interactors for the anti-GFP antibodies.
• **Results_2_GFP.xlsx** is the mass spectrometry report with annotated *N. crassa* proteins detected in the positive control (cytosolic sGFP from a crude extract). The listed proteins are considered potential sGFP interactors.
• **Results_3_G-TUB.xlsx, Results_4_MZT-1.xlsx, Results_5_APS2.xlsx**, and **Results_6_SPA10.xlsx** are reports with *N. crassa* proteins detected in the experiments. The identified proteins are potential interactors for γ-tubulin, MZT-1 (Mitotic Spindle Organizing Protein 1), APS-2 (anucleate primary sterigmata 2), and SPA10 (Septal Pore Associated Protein-10), respectively.
• **Filtered_TUB-GFP.xlsx, Filtered_MZT1-GFP.xlsx, Filtered_RFP-APS2.xlsx**, and **Filtered_SPA10-GFP.xlsx** files are the lists of identified proteins in the γ-tubulin, MZT-1, APS-2, and SPA10 experiments, less those shared proteins detected in controls (WT and GFP).

## 3 Experimental Design, Materials and Methods

**Fig. 1:**
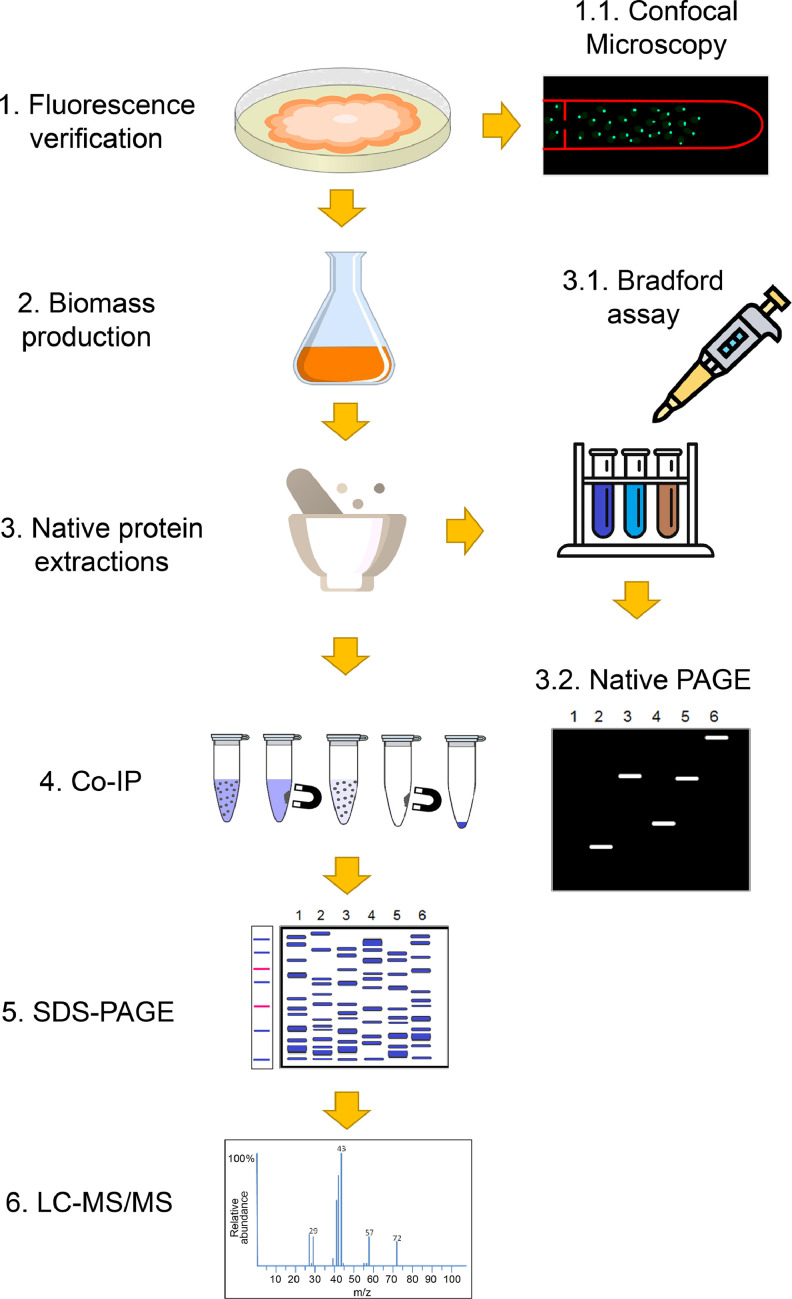
Experimental design to determine the protein-protein interactions. Before each experiment, we activate all strains on Vogel's minimal medium (VMM) agar to verify the viability and fluorescence by Confocal Fluorescent Microscopy. The biomass was obtained in liquid VMM at 30 °C, 150 rpm for 3 days (in the dark). The mycelia were macerated in liquid nitrogen and suspended in an extraction buffer (1:1 w/v). The crude extracts were clarified by centrifugation and filtration through a 0.22 µm membrane, and the Bradford method determined the protein amount. Also, the protein integrity and fluorescence were analyzed on native PAGE electrophoresis. For Co-IP assays, 1 mL of native protein extracts were incubated with 10–15 µL of GFP-Magnetic agarose beads overnight at 4 °C. After five gentle wash steps using a magnetic rack, the bounded proteins were isolated and eluted in a 2x SDS-Sample buffer. Pulldown proteins were analyzed in SDS-PAGE, and gel bands were cut and stored in sterile distilled water at 4 °C. Two biological replicates per experiment were sent for protein identification service by LC-MS/MS.

Protein-protein interactions of γ-tubulin (main MTOC component), MZT-1 (integral MTOC microprotein), APS-2 (MTOC and septal protein), and SPA-10 (pore septal protein) were pull-down by Co-IP and identified by LC-MS/MS using the experimental design depicted in Fig. 1. Here, a cytosolic sGFP expressing strain was included as a positive control and a WT strain as negative for background subtraction.

### 3.1 Culture media and reagents

#### 3.1.1 For culturing and microscopy

• Biotin stock solution (100 µg/mL): 5 mg biotin in 50 ml water or 50% ethanol.
• FM4–64 working solution (5 µM): 50 µL of dye stock solution (100 µg µM) in 950 µL ultra-pure water.
• Hygromycin B (commercial suspension 50 mg/mL).
• L-Histidine stock solution: 1.25 g Histidine in distilled water to a final volume of 50 mL and sterilized through a 0.45 µm membrane.
• Trace element solution: 5 g citric acid ·1H_2_O, 5 g ZnSO_4_ ·7H_2_O, 1 g Fe(NH_4_)_2_(SO_4_)_2_·6H_2_O, 0.25 g CuSO_4_·5H_2_O, 0.05 g MnSO_4_·1H_2_O, 0.05 g H_3_BO_3_ (anhydrous), 0.05 g Na_2_MoO_4_·2H_2_O, in 95 mL of distilled water. This solution can be stored at room temperature with 1 mL chloroform.
• VMM agar: 20 mL Vogel's 50X salts, 20 g sucrose, and 15 g agar per liter.
• VMM: 20 mL Vogel's 50X salts and 20 g sucrose per liter.
• Vogel's 50X salts: 150 g Na_3_ citrate ·5H_2_O, 126 g KNO_3_, 144 g (NH_4_)H_2_PO_4_, 80 g KH_2_ PO_4_, 10 g MgSO_4_ ·7H_2_O, 5 g CaCl_2_ ·2H_2_O, 5 mL trace element solution, and 2.5 mL of biotin stock solution (100 µg/mL) per liter. The solution can be stored at room temperature with a few milliliters of chloroform to preserve.

#### 3.1.2 For protein extraction and Co-IP

• Benzamidine stock solution (100 mM): 174.3 mg Benzamidine HCl in a final volume of 10 mL with sterile distilled water (used immediately before use).
• Bovine serum albumin (BSA) standards: 100 µg/mL, 200 µg/mL, 300 µg/mL, 400 µg/mL, 500 µg/mL, 600 µg/mL, 700 µg/mL, 800 µg/mL, 900 µg/mL, 1 mg/mL, 1.25 mg/mL, and 1500 mg/mL BSA.
• Bradford reagent: 100 mg/L Coomassie Brilliant Blue G-250, 50 mL/L ethanol, and 85% phosphoric acid.
• Commercial Protease inhibitor cocktail (AEBSF 100 mM, E-64 1.4 mM, Pepstatin A 2.2 mM 1,10-Phenanthroline 500 mM).
• Liquid nitrogen (LN_2_).
• Magnetic beads with anti-GFP antibodies (GFP-Trap® Magnetic Agarose, Chromotek).
• Medium flow filter paper (grade 1).
• PMSF stock solution (100 mM): 17.4 mg PMSF in isopropanol to a final volume of 10 mL, aliquoted and stored at −20 °C in the dark.
• Protein extraction buffer pH 7.5 (100 mM Tris–HCl, 100 mM NaCl, 0.5 mM EDTA, 30% glycerol, 1 mM PMSF, 1 mM Benzamidine, and protease inhibitor cocktail): 1.21 g Tris base, 0.58 g NaCl, 100 µL EDTA 0.5 M (pH 8.0), and 30 mL glycerol in 40 mL distilled water. Adjust pH with concentrated HCl solution, brought to a volume of 97 mL with distilled water, and sterilized through a 0.22 µM membrane. The working solution must be prepared at the moment by adding 10 µL PMSF (100 mM), 10 µL Benzamidine (100 mM), and 10 µL protease inhibitor cocktail per milliliter to be used.
• Protein wash buffer pH 7.5 (100 mM Tris–HCl, 100 mM NaCl, 0.5 mM EDTA, 1 mM PMSF, 1 mM Benzamidine, and protease inhibitor cocktail): 1.21 g Tris base, 0.58 g NaCl, and 100 µL EDTA 0.5 M (pH 8.0), all dissolved in 60 mL distilled water. Adjust the pH with concentrated HCl solution, bring it to 97 mL with distilled water, and pass through a 0.22 µM membrane to sterilize. Before use, an appropriate amount of buffer must be prepared by adding 10 µL PMSF (100 mM), 10 µL Benzamidine (100 mM), and 10 µL protease inhibitor cocktail per milliliter.

#### 3.1.3 Electrophoresis

• 0.5 M Tris–HCl (pH 6.8): 3.029 g Tris base in 30 mL distilled water. The pH was adjusted with concentrated HCl, brought to 50 mL with distilled water, and autoclaved (21 °C, 15 psi, 15 min).
• 1.5 M Tris–HCl (pH 8.8): 18.15 g Tris base suspended in 60 mL distilled water, pH-adjusted with concentrated HCl, and brought to 100 mL with distilled water. Autoclaved at 121 °C, 15 psi, for 15 min to sterilize.
• 10% Ammonium Persulfate (APS): 100 mg APS suspended in 900 µL distilled water (freshly prepared).
• 10% Sodium Dodecyl Sulfate (SDS): 1 g in 9 mL distilled water.
• 1X Electrophoresis buffer (25 mM Tris base, 192 mM glycine, 0.1% SDS, pH 8.6): mix 100 mL of 10X electrophoresis buffer and 900 mL sterile distilled water.
• 1X Native electrophoresis buffer (25 mM Tris base, 192 mM glycine, pH 8.6): 100 mL of concentrated native buffer and 900 mL of sterile distilled water.
• 2X Native sample buffer: 12.5 mL of 0.5 mM Tris–HCl (pH 6.8), 13.5 mL glycerol, 5 mg bromophenol blue in a final volume of 50 mL. Autoclaved (21 °C, 15 psi, 15 min) to sterilize.
• 2X Sample buffer: 12.5 mL of 0.5 mM Tris–HCl (pH 6.8), 10 mL glycerol, 2 g SDS, 5 mL 2-mercaptoethanol, and 5 mg bromophenol blue in a final volume of 50 mL. Filter sterilize through 0.22 µM membrane.
• 40% Acrylamide/Bis-acrylamide solution.
• Concentrated electrophoresis buffer (10X): 30.3 g of Tris base, 144.4 g of glycine, and 10 g of SDS for 1 L. Autoclaved (21 °C, 15 psi, 15 min) to sterilize.
• Concentrated native electrophoresis buffer (10X): 30.3 g Tris base and 144.4 g glycine for 1 L. Autoclaved (21 °C, 15 psi, 15 min) to sterilize.
• Distaining solution (500 mL): 250 mL methanol, 50 mL acetic acid glacial.
• Fixing solution (500 mL): 250 mL ethanol, 50 mL glacial acetic acid, and 200 mL distilled water.
• Pre-stained protein marker.
• Staining solution (500 mL): 150 mL methanol, 25 mL acetic acid glacial, 0.5 g Coomassie Blue R250, bring to 500 mL with distilled water.
• Tetramethylethylenediamine (TEMED), electrophoresis grade, ≥99%.

#### 3.1.4 For nano LC-MS/MS

• Acetonitrile, LC-MS Grade.
• DL-dithiothreitol ≥98% (HPLC).
• Formic acid, LC-MS Grade.
• Iodoacetamide ≥99% (NMR).
• Trypsin, LC-MS Grade.

#### 3.1.5 Instruments

• iBright™ CL1500 Imaging System (Thermo Fisher Scientific).
• Inverted confocal microscope Nikon ECLIPSE Ti-E Ti-E/B equipped with Spinning Disk CSU-200 × 1 Yokogawa.
• Mini-PROTEAN Tetra Handcast Systems (BioRad).
• SureBeads Magnetic Rack (Biorad).
• Ultimate 3000 nano UHPLC system coupled with a Q Exactive HF mass spectrometer (Thermo Fisher Scientific, USA) with an ESI nanospray source.

### 3.2 Strains phenotype confirmation

All strains described in Table 1 were first grown from conidia stocks in VMM agar plates, except the TRM-129-RR11 strain (expressing SPA-10-sGFP), which was grown in VMM agar supplemented with histidine (500 µg/mL) and hygromycin (300 µg/mL). The agar plates were incubated at 30 °C in the dark for 12–16 h. After that, a piece of agar with mycelium was cut to check the fluorescence by confocal microscopy by the inverted agar block method [1]. A drop of 5 µl FM4–64 dye (5 µM) was placed to visualize the cell membrane.

**Table 1:** List of *neurospora crassa* strains used in the experiments.

Strain	Genotype	Experiment	Fluorescent Protein Location	Ref.
FGSC 4200	*mat a wild type*	WT (control)	–	[2,3]
Cyt-sGFP	*mat A; his-3^+^::Pccg-1::sgfp^+^*	sGFP(control)	Cytosol	[4]
TRM01-RR01	*mat a; his-3^+^::Pccg-1-tbg-sgfp^+^*	γ-Tubulin-sGFP	SPB	[5]
MZT-1-GFP	*mat A; his-3^+^::Pccg-1-mzt-1-sgfp^+^*	MZT-1-sGFP	SPB	[5]
TRM-128-RR10	*mat A; his-3^+^::Pccg-1-drfp^+^-aps-2*	dRFP-APS-2	SPB and septa	[5]
TRM-129-RR11	*mat a; his-3::Pspa-10-spa-10-sgfp^+^; hph^+^*	SPA-10-sGFP	Septal pore	[5]

### 3.3 Biomass Production

Once the fluorescence was verified, all six strains were inoculated from conidia stocks (10 µL) in 50 mL of liquid VMM medium into 250 mL flasks. The flasks were covered with aluminum, and the cultures were incubated for three days at 30 °C with constant shaking (150 rpm). In the end, the biomass was collected on filter papers using a vacuum pump (without washing). Mycelia were stored at −80 °C or used immediately.

### 3.4 Protein Extraction

The mycelia were ground with liquid nitrogen in sterile mortars until a fine powder was obtained. The shredded biomass was weighed in a fresh 15 mL centrifuge tube, and ice-cold extraction buffer was added in 1:1 proportion (w/v) (*e.g.*, 1 mL buffer by 1 g biomass). The suspension was gently mixed by pipetting up and down to reduce the mechanical damage to a minimum. The insoluble material was collected by centrifugation at 12,000 rpm, 10 min, and 4 °C. After that, the supernatant was collected with a syringe and clarified through a 0.22 µm syringe filter. Aliquots of 100 µL were made to check protein quality and quantity, and the crude extracts were stored at −20 °C.

### 3.5 Protein Quantification and Native Page

**Fig. 2:**
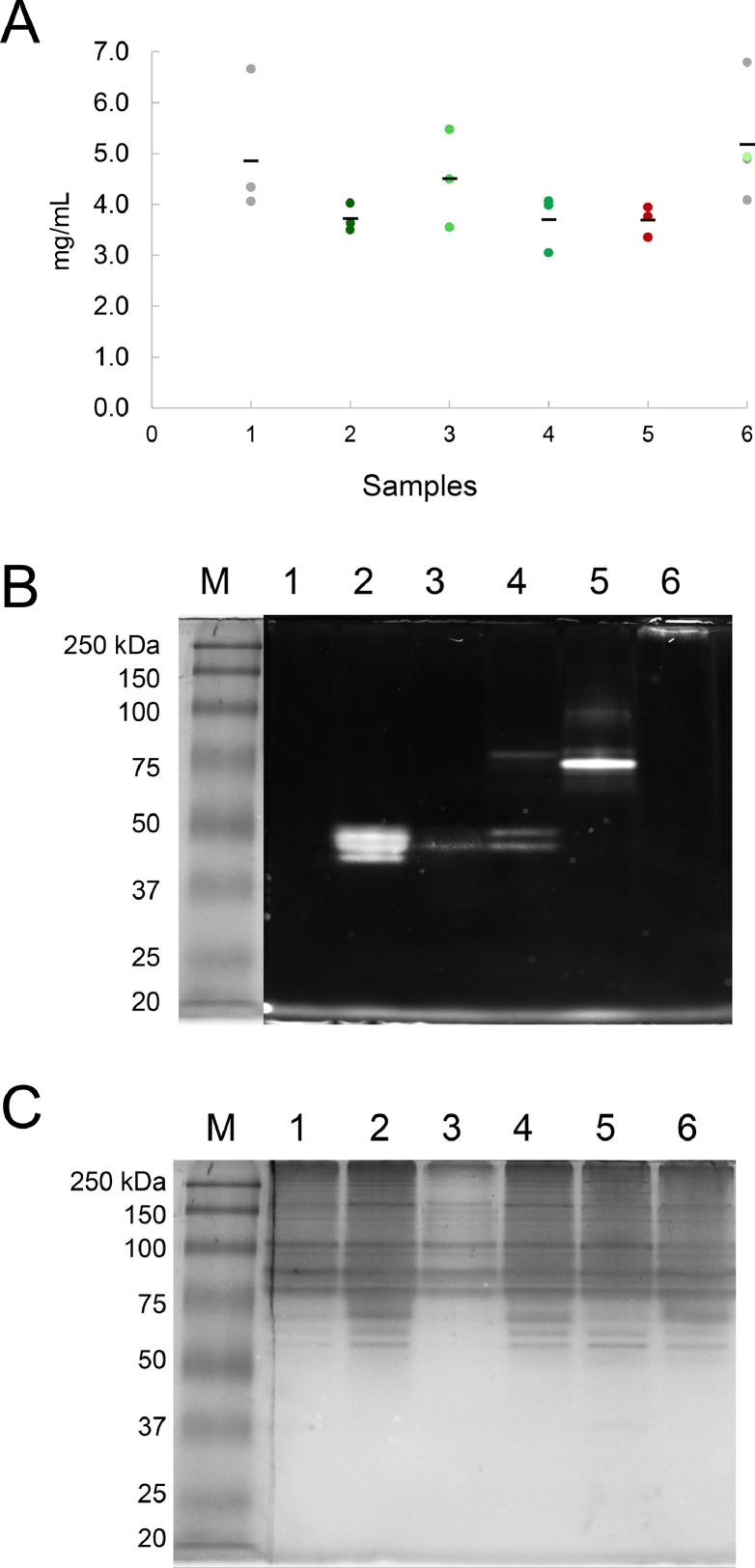
Native protein extractions for Co-IP experiments. A) Protein quantification by the Bradford method. Dots denote the values of each protein extraction, and scripts represent the arithmetic mean. Color code indicates fluorescence: red is the dRFP tag, green is the sGFP, and gray is no fluorescence registered by native PAGE or microscopy. B) Visualization of protein profiles in native PAGE 10% using a fluorescence filter. C) Coomassie blue staining after fluorescence visualization. Codes: M molecular weight marker, 1 WT, 2 sGFP, 3 γ-Tubulin-sGFP, 4 MZT-1-sGFP, 5 dRFP-APS2, 6 SPA10-sGFP.

The total extracted protein was quantified by the Bradford method (Fig. 2A), using BSA standards. A native polyacrylamide gel (10%) was prepared with 1.5 mL of 40% acrylamide/bisacrylamide, 2.6 mL Tris–HCl 1.5 M pH 8.8, 10 µL APS 10%, 10 µL SDS 10%, and 8 µL TEMED The protein profiles were analyzed on native PAGE 10% (prepared with 1.5 mL of 40% acrylamide/bisacrylamide, 2.6 mL Tris–HCl 1.5 M pH 8.8, 10 µL APS 10%, 10 µL SDS 10%, and 8 µL TEMED) according to [6]. Ten microliters of each sample were mixed with 10 µL 2X Native sample buffer and loaded using a prestained protein marker (Fig. 2B-C). Gels were first revealed with a green fluorescence filter (490–520 nm) to check the fluorescence. Afterward, gels were stained with Coomassie blue and visualized using the White Epi-LED (455–485 nm) in an iBright™ CL1500.

### 3.6 Co-IP

**Fig. 3:**
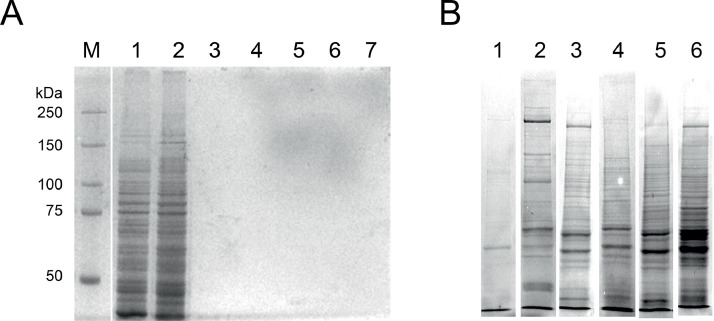
Electrophoresis of Co-IP experiments. A) Representative polyacrylamide gel following the Co-IP protocol (SPA10-sGFP experiment in SDS-PAGE 8%). Codes: M molecular weight marker, 1 input, 2 non-bound, 3–7 washes 1 to 5. B) Gel lanes with eluted proteins (bound) sent for LC-MS/MS analysis. Codes: 1 WT, 2 sGFP, 3: γ-Tubulin-sGFP, 4: MZT-1-sGFP, 5: dRPF-APS2, and 6 SPA10-sGFP.

Once the quantity and quality of the extracts were confirmed, the native proteins (input) were incubated with GFP-Magnetic agarose (GFP monoclonal nanobody/ VHH conjugated to magnetic agarose beads). The beads were previously washed and equilibrated in the extraction buffer according to the manufacturer's instructions. The binding was carried on 1.5 mL tubes with 1 mL of protein suspension (4–6 mg) and 10–15 µL bead slurry. The mixture was incubated overnight at 4 °C, under constant mixing (on a rotator) at 4 °C. Afterward, the beads were separated in the magnetic rack, and the supernatant was removed and stored at −20 °C (labeled as non-bound). The beads were washed four times with 500 µl ice-cold wash buffer. In each step, aliquots were stored at 4 °C (labeled as wash 1–4). Once more, the beads were suspended in 500 µl ice-cold wash buffer and transferred to a new 1.5 mL fresh tube, followed by magnetic separation and supernatant discard (wash 5) (Fig. 3A depicts the Co-IP steps in a representative experiment). The binding material was suspended in 50 µl 2x SDS-Sample buffer and boiled for 5 min at 95 °C. The tube was spun for 1 min at 12,000 rpm. The elution (labeled as bound) was stored at 4 °C.

### 3.7 SDS-PAGE

Two biological replicates of Co-IP experiments were analyzed in SDS-PAGE 8% stained with Coomassie blue [7]. Analyzing bounded proteins was carried out in a laminar flow hood, using sterile fresh-prepared solutions and sterile gel electrophoresis glasses. Gel casts and plastic materials were adequately cleaned and UV-sterilized for 15 min after use. The gel bands with bound samples (2 per experiment) were aseptically cut with a scalpel and transferred into 15 mL new centrifuge tubes with 5 mL distilled sterile water. The samples were sent in an ice pack to Creative Proteomics (Shirley, NY) for protein identification service (Fig. 3B shows one lane of each sample).

### 3.8 LC-MS/MS

#### 3.8.1 In-gel digestion

Each gel slice was cut into cubes of 1 mm3 and transferred to 1.5 mL microcentrifuge tubes with 1 mL 50 mM NH_4_HCO_3_/ acetonitrile (ACN) (1:1, v:v). After 30 min of incubation, the supernatant was removed, and the procedure was repeated until a complete discoloration. Next, 500 µL of ACN (Sigma-Aldrich) was added, and the mixture was incubated for 30 min. In the end, the gel pieces become opaque and stick together. ACN supernatant was removed, and the gel slices were rehydrated in 10 mM DL-dithiothreitol (DTT) (Sigma-Aldrich), followed by incubation at 56 °C for 1 h. The DTT was carefully removed, and 500 µL of ACN was added and incubated for 10 min at room temperature. The ACN was carefully removed, and 50 mM iodoacetamide (IAA) (Sigma-Aldrich) was poured until the gel slice was covered entirely. Then, an incubation period of 30 min at room temperature in the dark. The IAA was removed, and the gel slices were incubated in 500 µL of ACN for 10 min at room temperature. Next, the ACN solution was discarded, and a Rapid-Digestion Trypsin solution (Promega) was poured to cover the gel slices. The tube was incubated on ice for 45 min. If needed, more digestion solution was added if the gel pieces absorbed all the initial solution. The excess digestion solution was removed and added 5–20 µL of 50 mM NH_4_HCO_3_ was to keep the gel pieces wet during enzymatic digestion. The excess digestion solution was removed, and 5–20 µL of 50 mM NH_4_HCO_3_ was used to keep the gel pieces wet during enzymatic digestion. The supernatant was transferred into a fresh 1.5 mL microcentrifuge tube where 50 mM ammonium bicarbonate/acetonitrile solution (1:2, v/v) was added to cover gel slices. An incubation period of 1 h was set at 37 °C. The solution was transferred to a new 1.5 mL microcentrifuge tube to lyophilize the extracted peptides to near dryness. Peptides were resuspended in 20 µL of 0.1% formic acid before LC-MS/MS analysis.

#### 3.8.2 Nano-Liquid chromatography

For Liquid Chromatography, 1 µg of the sample was loaded and subject to a linear gradient from 2 to 8% buffer B for 3 min, then from 8% to 20% buffer B for 50 min; next, from 20% to 40% buffer B in 43 min; and finally, from 40% to 90% buffer B in 4 min, in the mobile phase A. The total flow rate was 250 nL/min.

#### 3.8.3 Mass spectrometry

The full scan was performed between 300 and 1650 *m/z* at the resolution 60,000 at 200 *m/z*. The automatic gain control target for the full scan was set to 3e6. The MS/MS scan was operated in Top 20 mode using the following settings: resolution 15,000 at 200 *m/z*; automatic gain control target 1e5; maximum injection time 19 ms; normalized collision energy at 28%; isolation window of 1.4 Th; charge sate exclusion: unassigned, 1, > 6; dynamic exclusion 30 s.

#### 3.8.4 Data Analysis

The six raw MS files were analyzed and searched against the *N. crassa* protein database based on the species of the samples using MaxQuant (1.6.0.1) [8]. The detailed protein and peptide identification information was sent in an Excel file.

Approximately two-thirds of the identified proteins were shared with those listed in the controls (WT and sGFP). To subtract the background and potential spurious results, all the proteins listed in the controls were eliminated from the experiment. After that, more than 500 proteins remained for each experiment (Filtered lists).

## Limitations

The methods described here apply to the Co-IP technique using magnetic agarose pre-immobilized with anti-GFP antibodies (host alpaca), which lacks heavy and light antibody chains. Hence, it is not suitable for a free antibody IP approach. Also, a higher background is expected when using whole anti-GFP antibodies (with heavy and light antibody chains).

## Ethics Statement

This work does not involve human subjects, animal experiments, or data collected from social media platforms. The authors have read and declare that the manuscript meets all the ethical requirements for publication (according to https://www.elsevier.com/authors/journal-authors/policies-and-ethics).

## CRediT authorship contribution statement

**Astrid N. Espino-Vázquez:** Methodology, Investigation, Data curation, Formal analysis, Writing – original draft. **Rosa Ramírez Cota:** Methodology, Investigation. **Olga Alicia Callejas-Negrete:** Resources, Methodology, Writing – review & editing. **Reinhard Fischer:** Funding acquisition, Writing – review & editing. **Rosa R. Mouriño-Pérez:** Conceptualization, Supervision, Funding acquisition, Project administration, Writing – review & editing.

## Data Availability

Results Co-IP (Original data) (Mendeley Data)
